# Prognostic analysis of E2F transcription factors *E2F1* and *E2F3* in four independent pediatric neuroblastoma cohorts

**DOI:** 10.1186/s12887-022-03424-w

**Published:** 2022-06-29

**Authors:** Haiwei Wang, Xinrui Wang, Liangpu Xu, Ji Zhang

**Affiliations:** 1 Fujian Maternity and Child Health Hospital, Fujian Medical University, Fuzhou, 350001 Fujian China; 2 Rui-Jin Hospital Affiliated to Shanghai Jiao Tong University School of Medicine, Shanghai, 200025 China

**Keywords:** Pediatric neuroblastoma, E2F transcription factor, *E2F1*, *E2F3*, *MYCN* amplification, Age

## Abstract

**Background:**

Previously, we had analyzed the prognosis of E2F transcription factors across adult tumor types. However, the expressions and prognosis of E2F transcription factors in pediatric neuroblastoma have not yet been fully studied.

**Methods:**

The prognosis of E2F transcription factors was determined in four independent pediatric neuroblastoma cohorts from Therapeutically Applicable Research to Generate Effective Treatments (TARGET), Gene Expression Omnibus (GEO) and European ArrayExpres datasets using Kaplan–Meier and cox regression analysis.

**Results:**

E2F regulated gene set was associated with the event free survival and the overall survival of neuroblastoma. *E2F1* and *E2F3* were prognostic factors in all four independent pediatric neuroblastoma cohorts. Over-expressions of *E2F1* or *E2F3* were correlated with the shorted event free survival and overall survival of neuroblastoma. Expression levels of *E2F1* and *E2F3* were higher in neuroblastoma patients with *MYCN* amplification or age at diagnosis ≥ 18 months. Moreover, the prognostic significance of *E2F1* or *E2F3* in neuroblastoma was independent of *MYCN* amplification and age of diagnosis. Combinations of *E2F1*, *E2F3* with *MYCN* amplification or age of diagnosis achieved better prognosis of neuroblastoma. Identification of 234 genes were associated with *E2F1* and *E2F3* expressions in neuroblastoma and those genes were significantly enriched in cell cycle signaling pathway. Also, higher scores of cell cycle signaling pathway were correlated with the adverse prognosis of neuroblastoma.

**Conclusions:**

E2F transcription factors *E2F1* and *E2F3* were prognostic makers of neuroblastoma.

**Supplementary Information:**

The online version contains supplementary material available at 10.1186/s12887-022-03424-w.

## Introduction

E2F transcription factors are referring *E2F1* to *E2F8* eight genes [1]. The E2F family genes are critical to the development of cancer by regulations of DNA replication and cell cycle progression [2–4]. Expression levels of E2F family genes were associated with the clinical outcomes of bladder cancer, prostate cancer, lung cancer, colon cancer or breast cancer [5–7]. Previously, using The Cancer Genome Atlas datasets, we had analyzed the expressions and prognosis of E2F transcription factors across different tumor types [8]. Our results suggested the unfavorable prognostic effects of E2F transcription factors in liver hepatocellular carcinoma (LIHC) and lung adenocarcinoma (LUAD). However, the analysis was focusing on adult tumor types. The expressions and prognosis of E2F transcription factors in pediatric cancer types, particular in pediatric neuroblastoma are unclear.

Neuroblastoma is a malignant pediatric disease with poor prognosis [9, 10]. *MYCN* amplification is detected in approximate 25% neuroblastoma patients [11]. *MYCN* amplification [12] and *MYCN* high expression [13] represent adverse prognostic factors in neuroblastoma. E2F transcription factors are involved in the development of neuroblastoma by regulating *MYCN* expression [14]. Our previous results showed that *E2F1* was a target of *MYCN* amplification and associated with the poor overall survival of neuroblastoma [15]. Moreover, *MYCN* amplification induced *E2F5* expression to promote neuroblastoma progression through regulation of cell cycle pathway [16]. The higher expression levels of *E2F3* were associated with the worse clinical outcomes of stage 4S neuroblastoma [17]. However, the expressions and prognosis of E2F transcription factors have not yet been studied in neuroblastoma in a comprehensive manner.

With the improvements of technologies, multiple neuroblastoma cohorts had been studied in transcriptional analysis. Previously, using integrated neuroblastoma cohorts, we analyzed the transcriptional features of neuroblastoma associated with *MYCN* amplification [15] and age at diagnosis ≥ 18 months [18]. Here, using four independent neuroblastoma cohorts from Therapeutically Applicable Research to Generate Effective Treatments (TARGET), Gene Expression Omnibus (GEO) and European ArrayExpres datasets, we studied the expressions and prognosis of E2F transcription factors in neuroblastoma. Our results suggested that *E2F1* and *E2F3* were prognostic makers of neuroblastoma independent of *MYCN* amplification and age of diagnosis.

## Materials and methods

### Data collection and processing

TARGET datasets were downloaded from https://ocg.cancer.gov/ [19]. GSE16476 [20–22] and GSE85047 [23] was downloaded from the GEO website (www.ncbi.nlm.nih.gov/geo). E-MTAB-1781 dataset [24] were downloaded from https://www.ebi.ac.uk/arrayexpress/ website. Neuroblastoma patients with event free survival or overall survival were selected for further studies. All the data was processed using R software. The matrix files were annotated by corresponding platforms. Averaged expression values of same gene symbol were used for further studies.

### Single sample Gene Set Enrichment Analysis (ssGSEA)

E2F_01 associated gene set (derived from c3.tft.v7.2.symbols gene sets) and cell cycle signaling pathway associated gene set (derived from c2.cp.kegg.v7.2.symbols gene sets) were downloaded from GSEA website. The scores of E2F_01 and cell cycle signaling pathway were determined using ssGSEA assay “GSVA” package in R software [25]. “GSVA” is a non-parametric, unsupervised method for estimating variation of gene set enrichment through the expression dataset, thereby evaluating the scores of pathways or transcription factors in each sample.

### Univariable and multivariable cox regression analysis

Univariable and multivariable cox regression analysis were carried out using “survival” and “survminer” packages “coxph” method in R software. The forest plots were generated using “forestplot” and “ggforest” packages in R software. The hazard ratio (HR) and *P* values were determined using cox regression survival analysis.

### Kaplan–Meier survival analysis

Kaplan–Meier plots were created using “survival” and “survminer” packages in R software. Pediatric neuroblastoma patients were divided into “high” or “low” sub-groups based on the optimal cutoff points using “survminer” package “surv_cutpoint” method. “surv_cutpoint” is an outcome-oriented method by calculating all possible cutoff values between the lower and upper sub-groups. Cutoff points that were most significantly associated with the clinical outcomes were selected as the best cutoff points. *P* values were determined using log-rank test in “survival” package.

### Risk score plot

The risk score plots were generated using “ggrisk” and “rms” packages in R software. The risk score was calculated based on the cox regression in “survival” package by summing up the variables in the cox model weighted by the corresponding regression coefficients. The cutoff was determined by the median of the risk score.

### Venn diagram

The overlapping of different gene lists was carried out using in TBtools software (https://github.com/CJ-Chen/TBtools/releases) [26]. TBtools was a Toolkit for integrating various data.

### Heatmap presentation

The expression features of the genes associated with *E2F1* and *E2F3* expressions in neuroblastoma were clustered using “pheatmap” package in R software. The “average” method and “correlation” were used to determine the clustering scale and clustering distance, respectively.

### Kyoto Encyclopedia of Genes and Genomes (KEGG) signaling pathway and transcription factor enrichment analysis

*E2F1* and *E2F3* associated KEGG signaling pathways and transcription factors were determined using The Database for Annotation, Visualization and Integrated Discovery (DAVID) website (https://david.ncifcrf.gov) [27, 28]. The False Discovery Rate was used for multiple hypothesis testing. Signaling pathways or transcription factors with *P* values < 0.05 were significantly enriched.

### Statistical analysis

Statistical analysis was performed using the two tails paired student’s t test in GraphPad Prism software. *P* value < 0.05 was chosen to be significantly different.

## Results

### E2F target gene set is associated with the poor prognosis of neuroblastoma

In order to determine the expressions and prognosis of E2F family genes in neuroblastoma, expression profiles of 1102 pediatric neuroblastoma patients from four independent datasets, including TARGET, GSE16476, GSE85047 and E-MTAB-1781 datasets were collected (supplementary Fig. 1a). Age of neuroblastoma diagnosis, percentage of MYCN amplified neuroblastoma and platforms of the four datasets were significantly different. TARGET dataset had the oldest age of neuroblastoma diagnosis and the highest percentage of MYCN amplification. Also, compared with other datasets, neuroblastoma patients in TARGET dataset had the most unfavorable event free survival and overall survival (supplementary Fig. 1b). Because of the large discrepancy between databases, we studied the expressions and prognosis of E2F transcription factors in each pediatric neuroblastoma cohort.

**Fig. 1:**
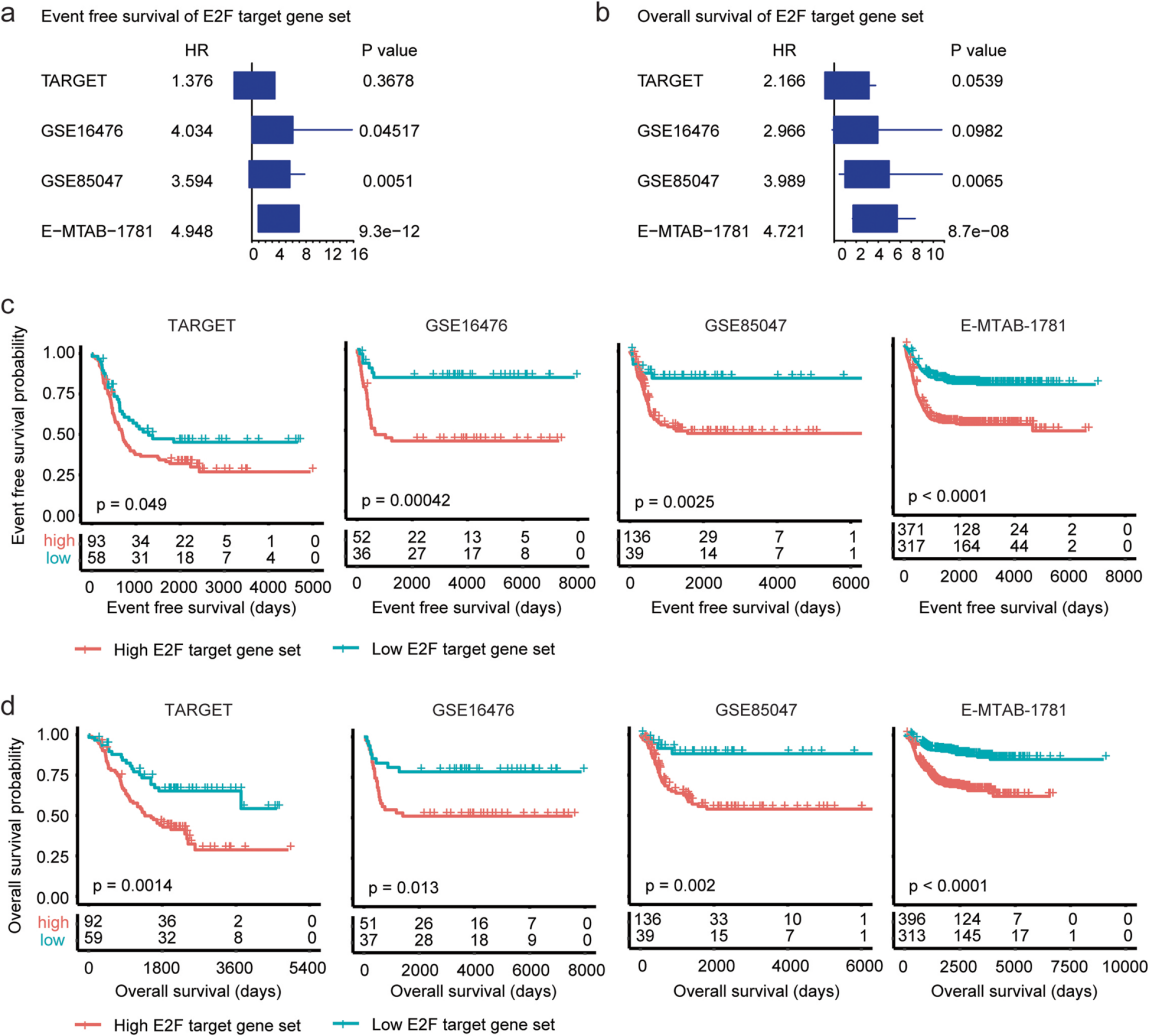
Higher scores of E2F target gene set are associated with the worse prognosis of neuroblastoma. **a** Forest plot showed the associations of E2F target gene set with the neuroblastoma event free survival in TARGET, GSE16476, GSE85047 and E-MTAB-1781 datasets. **b** Forest plot showed the associations of E2F target gene set with the neuroblastoma overall survival. **c** The Kaplan–Meier curves showed the different event free survival of pediatric neuroblastoma patients with higher or lower scores of E2F target gene set in TARGET, GSE16476, GSE85047 and E-MTAB-1781 datasets. **d** Different overall survival of pediatric neuroblastoma patients with higher or lower scores of E2F target gene set

First, the score of E2F target gene set was calculated using ssGSEA and the prognosis of E2F target gene set was determined using univariable cox regression. In GSE16476, GSE85047 and E-MTAB-1781 datasets, E2F target gene set was significantly correlated with the event free survival of neuroblastoma (Fig. 1a). Moreover, E2F target gene set was significantly associated with the overall survival of neuroblastoma in GSE85047 and E-MTAB-1781 datasets (Fig. 1b).

Furthermore, the Kaplan–Meier survival analysis confirmed the prognosis of E2F target gene set in neuroblastoma. Neuroblastoma patients with lower scores of E2F target gene set had prolonged event free survival in TARGET, GSE16476, GSE85047 and E-MTAB-1781 datasets (Fig. 1c). Higher scores of E2F target gene set were also associated with the worse overall survival of neuroblastoma in TARGET, GSE16476, GSE85047 and E-MTAB-1781 datasets (Fig. 1d).

### Prognostic effects of the E2F transcription factors in neuroblastoma

**Fig. 2:**
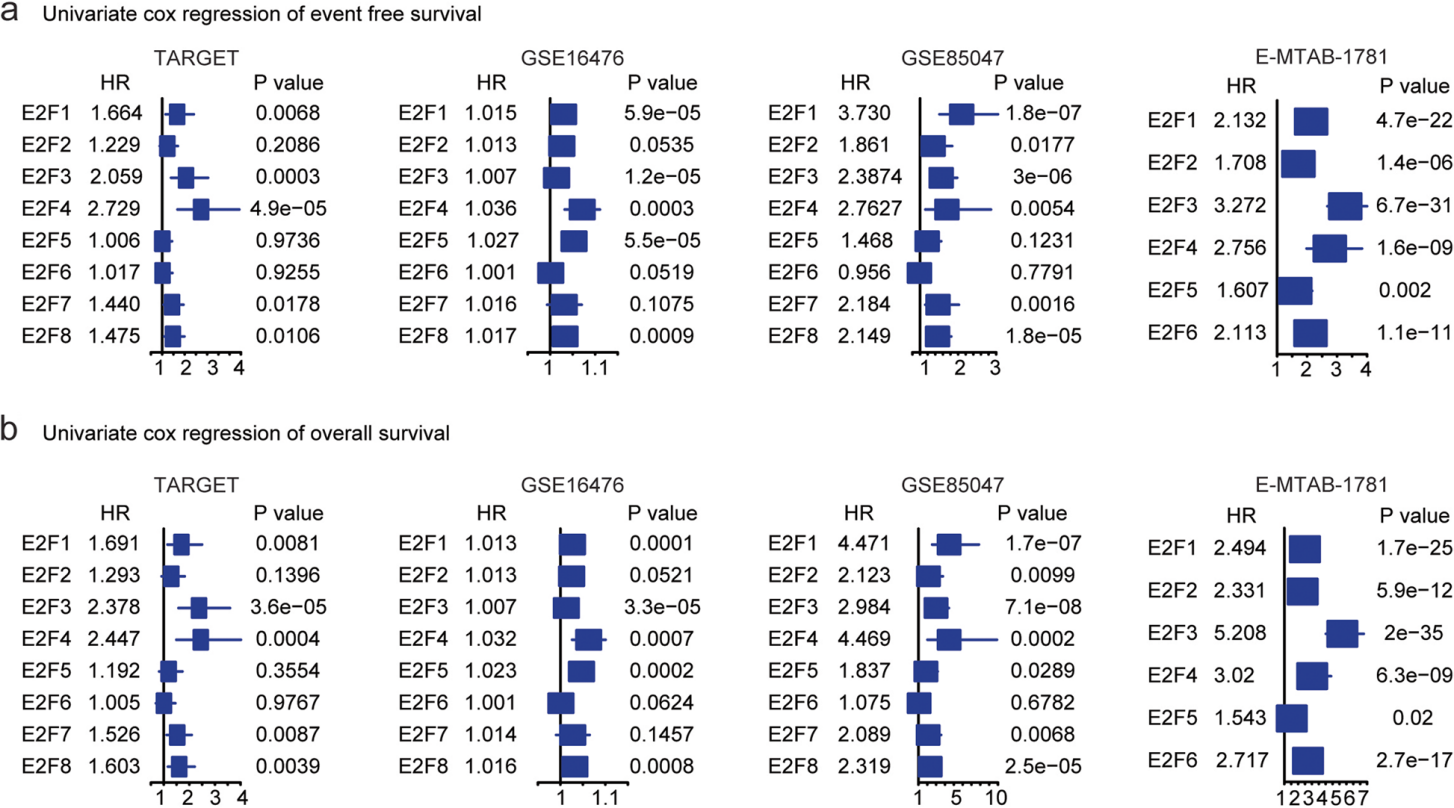
Prognostic effects of the E2F transcription factors in neuroblastoma. **a** Forest plots showed the associations of E2F transcription factors with the neuroblastoma event free survival in TARGET, GSE16476, GSE85047 and E-MTAB-1781 datasets. **b** Forest plots showed the associations of E2F transcription factors with the neuroblastoma overall survival

E2F transcription factors include eight numbers [1]. The prognosis of each E2F gene was investigated in TARGET, GSE16476, GSE85047 and E-MTAB-1781 datasets using univariable cox regression assay. E2F transcription factors *E2F1*, *E2F3*, *E2F7* and *E2F8* were all associated with the event free survival of neuroblastoma in TARGET, GSE16476 and GSE85047 datasets (Fig. 2a). In E-MTAB-1781 dataset, the expressions of *E2F7* and *E2F8* were not detected, while, *E2F1* and *E2F3* were also associated with the event free survival of neuroblastoma in E-MTAB-1781 datasets (Fig. 2a). Moreover, *E2F2* was correlated with the event free survival of neuroblastoma in GSE85047 and E-MTAB-1781 datasets, but not in TARGET and GSE16476 datasets (Fig. 2a). *E2F4* was associated with the event free survival of neuroblastoma in TARGET, GSE16476, GSE85047 and E-MTAB-1781 datasets (Fig. 2a). *E2F5* had prognostic significance in GSE85047 and E-MTAB-1781 datasets (Fig. 2a) and *E2F6* had prognostic significance in E-MTAB-1781 datasets (Fig. 2a).

Furthermore, *E2F1* and *E2F3* were both significantly associated with the overall survival of neuroblastoma in TARGET, GSE16476, GSE85047 and E-MTAB-1781 datasets (Fig. 2b). *E2F2* was associated with the overall survival of neuroblastoma in GSE85047 and E-MTAB-1781 datasets, but not in TARGET and GSE16476 datasets (Fig. 2b). *E2F8* was associated with the overall survival of neuroblastoma in TARGET, GSE16476 and GSE85047 datasets (Fig. 2b). *E2F4* was associated with the overall survival of neuroblastoma in TARGET, GSE16476, GSE85047 and E-MTAB-1781 datasets (Fig. 2b). Combined the univariable cox regression assay in four independent datasets, *E2F1* and *E2F3* were most significantly correlated with the event free survival and overall survival of neuroblastoma.

### Higher expression levels of *E2F1* or *E2F3* are associated with the worse prognosis of neuroblastoma

**Fig. 3:**
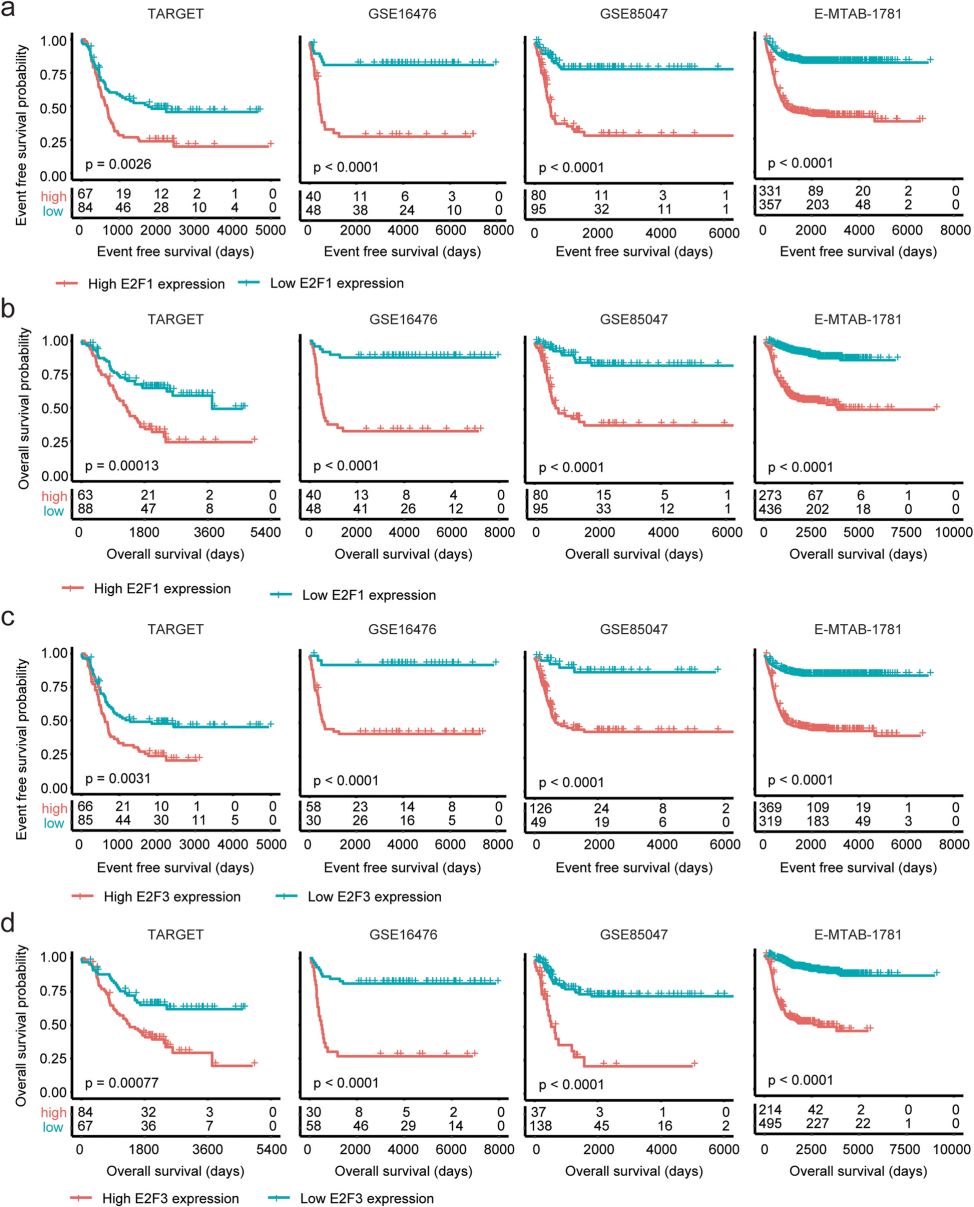
Higher expression levels of *E2F1* or *E2F3* are associated with the worse prognosis of neuroblastoma. **a** The Kaplan–Meier curves showed the event free survival of pediatric neuroblastoma patients with *E2F1* higher expressions or *E2F1* lower expressions in TARGET, GSE16476, GSE85047 and E-MTAB-1781 datasets. **b** Overall survival of pediatric neuroblastoma patients with *E2F1* higher expressions or *E2F1* lower expressions. **c** The Kaplan–Meier curves showed the event free survival of pediatric neuroblastoma patients with *E2F3* higher expressions or *E2F3* lower expressions in TARGET, GSE16476, GSE85047 and E-MTAB-1781 datasets. **d** Overall survival of pediatric neuroblastoma patients with *E2F3* higher expressions or *E2F3* lower expressions

The prognosis of *E2F1* and *E2F3* transcription factor was further determined using Kaplan–Meier survival analysis. Previously, using TARGET and GSE85047 datasets, we had shown that transcription factor *E2F1* was associated with the poor overall survival of neuroblastoma [15]. Similar with the results from TARGET and GSE85047 datasets, neuroblastoma patients with higher expression levels of *E2F1* were associated with the lower event free survival in GSE16476 and E-MTAB-1781 datasets (Fig. 3a). Also, compared with neuroblastoma patients with higher expression levels of *E2F1*, neuroblastoma patients with lower expression levels of *E2F1* had significantly prolonged overall survival in GSE16476 and E-MTAB-1781 datasets (Fig. 3b).

Neuroblastoma patients with higher expression levels of *E2F3* were also had lower event free survival in TARGET, GSE16476, GSE85047 and E-MTAB-1781 datasets, compared with neuroblastoma patients with lower expression levels of *E2F3* (Fig. 3c). Moreover, *E2F3* was associated with the overall survival of neuroblastoma in TARGET, GSE16476, GSE85047 and E-MTAB-1781 datasets. Compared with neuroblastoma patients with higher expression levels of *E2F3*, neuroblastoma patients with lower expression levels of *E2F3* had significantly prolonged overall survival (Fig. 3d). Results from both cox regression assay and Kaplan–Meier survival analysis suggested that *E2F1* and *E2F3* transcription factors were correlated with the event free survival and overall survival of neuroblastoma.

### Expression levels of *E2F1* or *E2F3* are associated with* MYCN* amplification or age of diagnosis in neuroblastoma

**Fig. 4:**
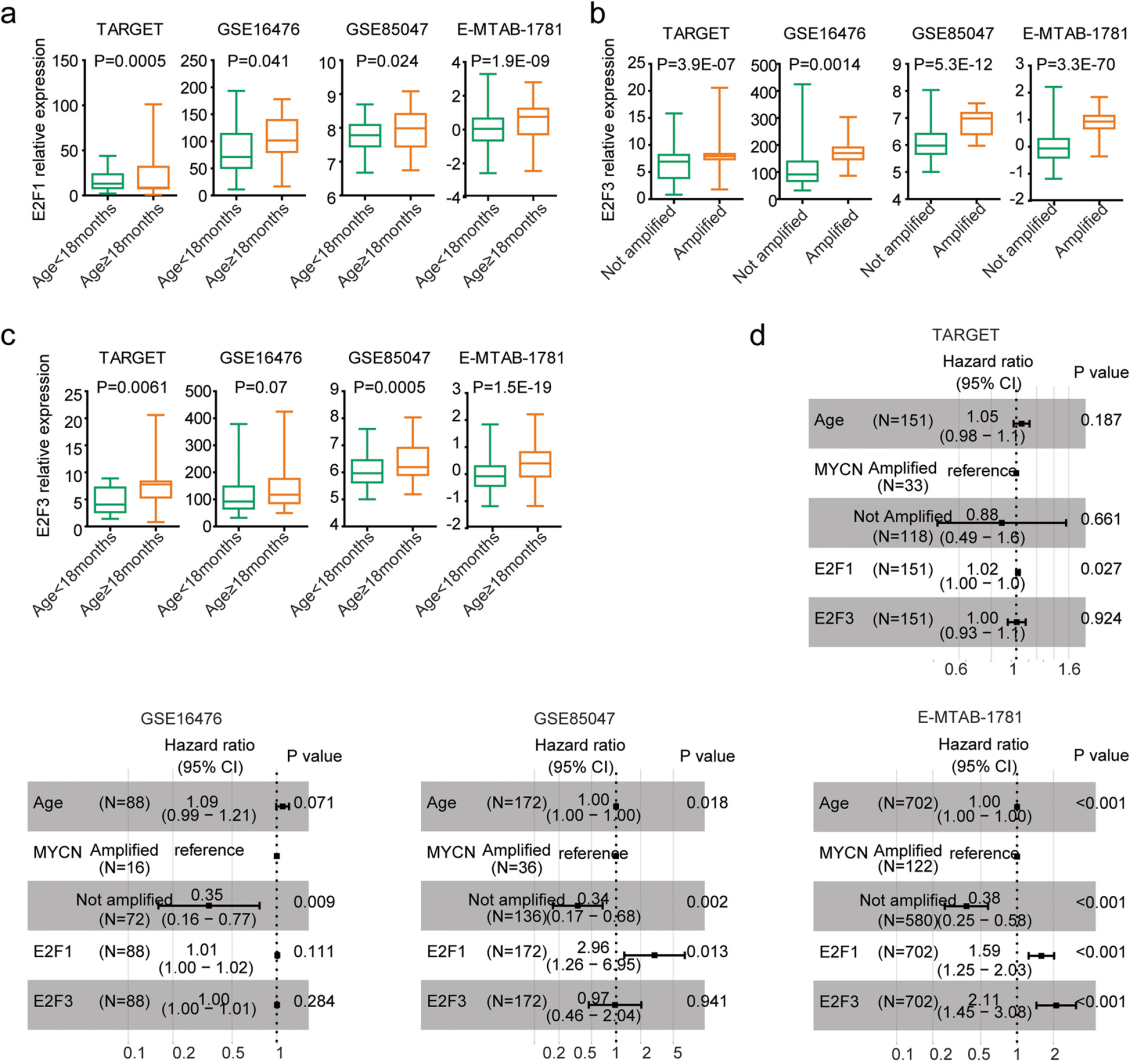
*E2F1* or *E2F3* are associated with *MYCN* amplification or age of diagnosis in neuroblastoma. **a** Box plots showed the relative *E2F1* expression levels in pediatric neuroblastoma patients with age of diagnosis ≥ 18 month or < 18 months in TARGET, GSE16476, GSE85047 and E-MTAB-1781 datasets. **b** The relative *E2F3* expression levels in pediatric neuroblastoma patients with or without *MYCN* amplification. **c** The relative *E2F3* expression levels in pediatric neuroblastoma patients with age of diagnosis ≥ 18 month or < 18 months. **d** Forest plots showed the associations of *E2F1* expression, *E2F3* expression, *MYCN* amplification and age of diagnosis with the clinical overall survival of pediatric neuroblastoma patients in TARGET, GSE16476, GSE85047 and E-MTAB-1781 datasets. Hazard ratio (HR) and *P* values were determined using multivariable cox regression assay

*MYCN* amplification and age of diagnosis were critical determiners of the clinical outcomes of pediatric neuroblastoma [12]. Previously, our results showed that *E2F1* is up-regulated by *MYCN* amplification [15]. However, the relationships of *E2F1* expression and age of diagnosis in neuroblastoma are unclear. In TARGET, GSE16476, GSE85047 and E-MTAB-1781 datasets, the *E2F1* expression levels were higher in neuroblastoma patients with age at diagnosis ≥ 18 months than neuroblastoma patients with age at diagnosis < 18 months (Fig. 4a).

We also determined the relationships of *E2F3* expression, *MYCN* amplification and age of diagnosis in neuroblastoma patients. *E2F3* expression levels were higher in *MYCN* amplified neuroblastoma patients in TARGET, GSE16476, GSE85047 and E-MTAB-1781 datasets, compared with neuroblastoma patients without *MYCN* amplification (Fig. 4b). Furthermore, in TARGET, GSE85047 and E-MTAB-1781 datasets, the expression levels of *E2F3* were also higher in neuroblastoma patients with age at diagnosis ≥ 18 months than neuroblastoma patients with age at diagnosis < 18 months (Fig. 4c).

### *E2F1* and *E2F3* are prognostic makers of neuroblastoma independent of *MYCN* amplification and age of diagnosis

We then assessed the associations of age of diagnosis, *MYCN* amplification and *E2F1* expression and *E2F3* expression in the prediction of the overall survival of neuroblastoma using multivariable cox regression assay. Age of diagnosis and *MYCN* amplification were independent prognostic factors of pediatric neuroblastoma in GSE85047 and E-MTAB-1781 datasets (Fig. 4d). *MYCN* amplification was also an independent prognostic factor in GSE16476 dataset (Fig. 4d).

Moreover, *E2F1* was a prognostic maker of neuroblastoma independent of *MYCN* amplification, age of diagnosis and *E2F3* expression in TARGET, GSE85047 and E-MTAB-1781 datasets (Fig. 4d). *E2F3* was also a prognostic maker of neuroblastoma independent of *MYCN* amplification, age of diagnosis and *E2F1* expression in E-MTAB-1781 datasets (Fig. 4d).

### Additively prognostic effects of *E2F1* with *MYCN* amplification or age of diagnosis in neuroblastoma

**Fig. 5:**
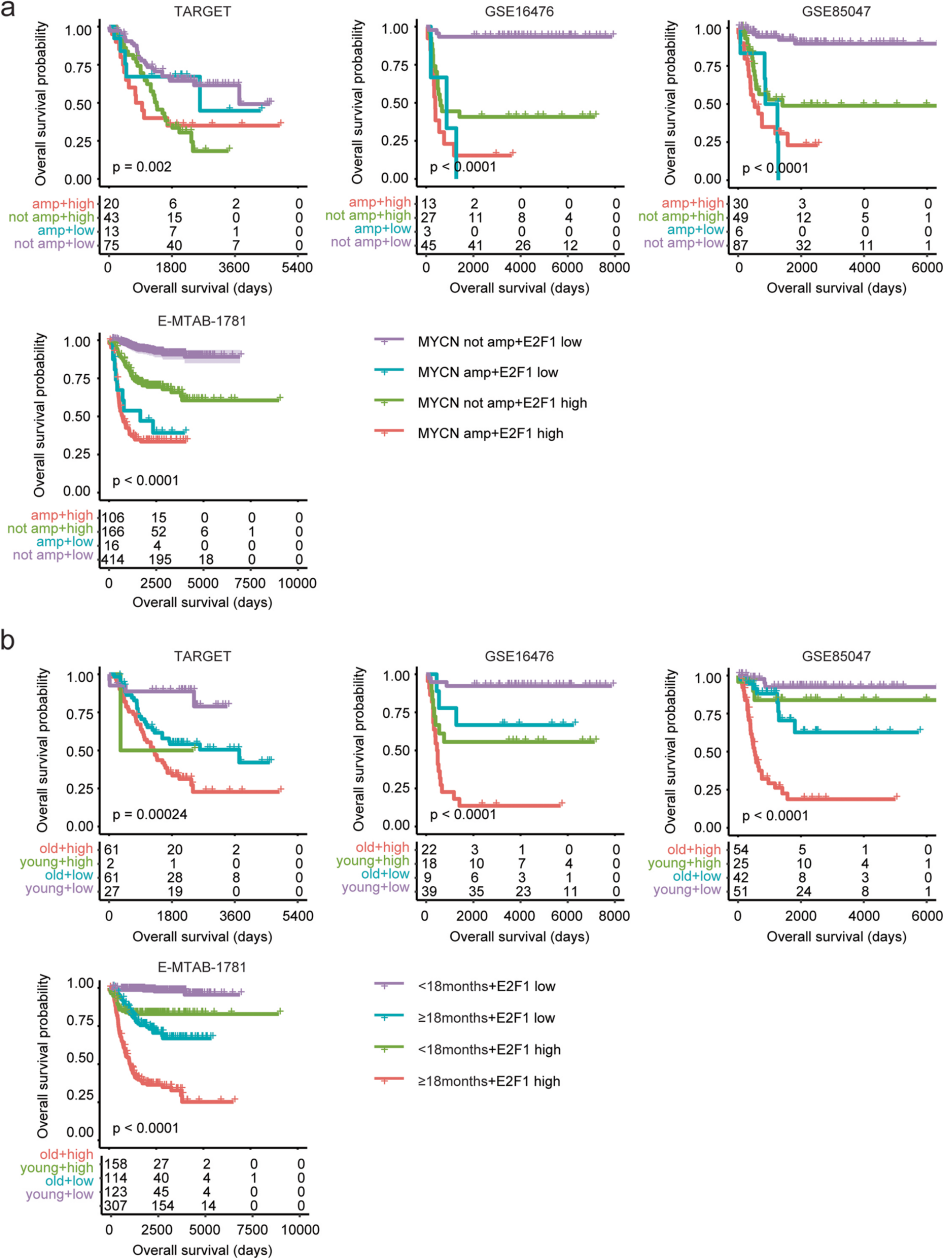
Additively prognostic effects of *E2F1* with *MYCN* amplification or age of diagnosis in neuroblastoma. **a** Pediatric neuroblastoma patients were divided into four sub-groups based on the expression levels of *E2F1* and *MYCN* amplification. Different overall survival in each sub-group of pediatric neuroblastoma was determined using the Kaplan–Meier survival analysis in TARGET, GSE16476, GSE85047 and E-MTAB-1781 datasets. **b** Pediatric neuroblastoma patients were divided into four sub-groups based on the expression levels of *E2F1* and age of diagnosis. Different overall survival in each sub-group of pediatric neuroblastoma was determined

Since, *E2F1* and *E2F3* were prognostic makers of neuroblastoma independent of *MYCN* amplification and age of diagnosis, the combinations of *E2F1* or *E2F3* with *MYCN* amplification or age of diagnosis could achieve better prognostic effects in pediatric neuroblastoma patients. Based on the expression levels of *E2F1* and *MYCN* amplification, pediatric neuroblastoma patients were divided into four sub-groups. Pediatric neuroblastoma patients without *MYCN* amplification and with lower *E2F1* expression levels had significantly longer event free survival and overall survival in GSE16476, GSE85047 and E-MTAB-1781 datasets (Fig. 5a and supplementary Fig. 2a).

Moreover, pediatric neuroblastoma patients with age at diagnosis < 18 months and with lower *E2F1* expression levels had the best clinical event free survival and overall survival than other sub-groups in TARGET, GSE16476, GSE85047 and E-MTAB-1781 datasets (Fig. 5b and supplementary Fig. 2b). On the contrary, pediatric neuroblastoma patients with age at diagnosis ≥ 18 months and with higher *E2F1* expression levels had the worst event free survival and overall survival than other sub-groups in TARGET, GSE16476, GSE85047 and E-MTAB-1781 datasets (Fig. 5b and supplementary Fig. 2b).

### Additively prognostic effects of *E2F3* with *MYCN* amplification or age of diagnosis in neuroblastoma

**Fig. 6:**
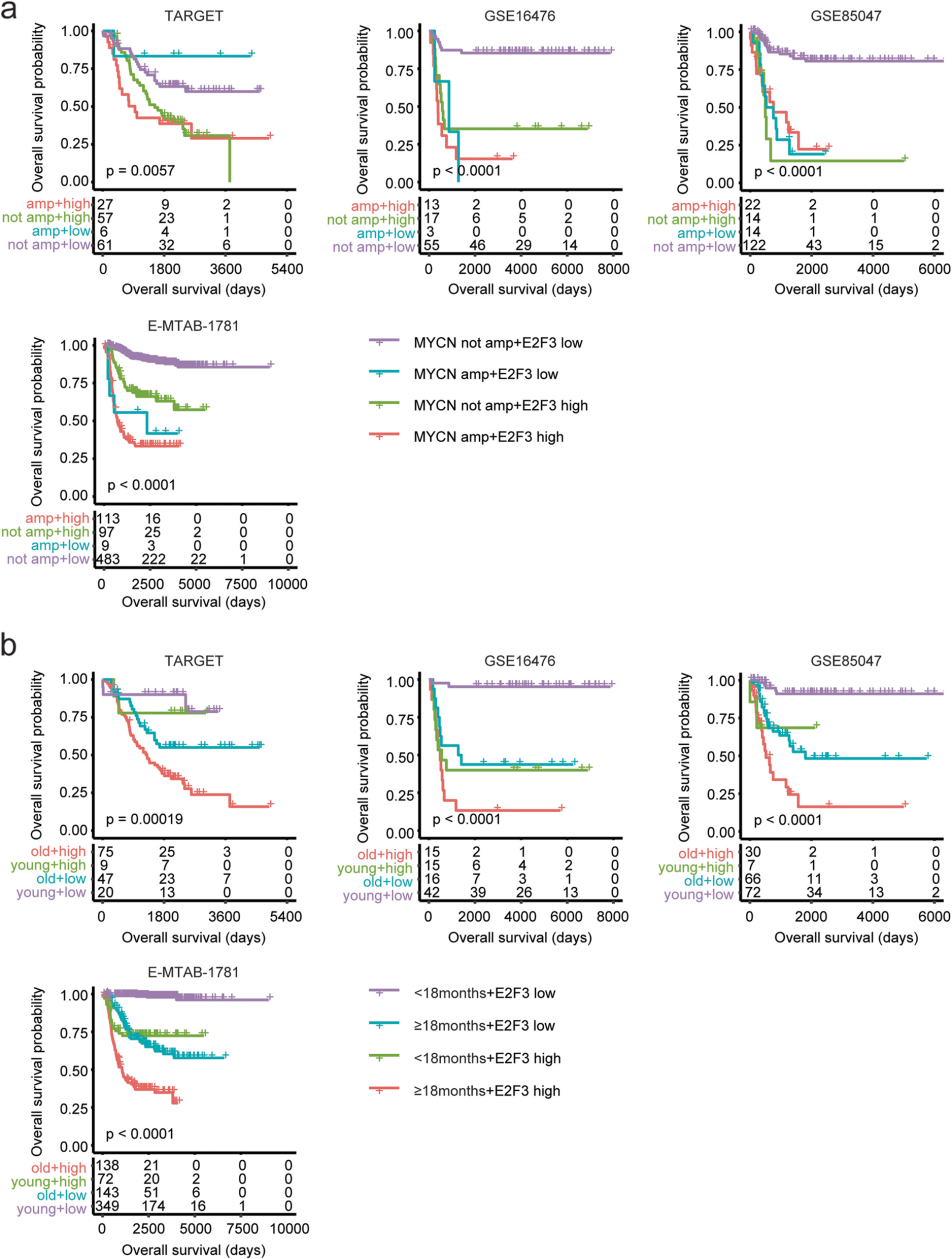
Additively prognostic effects of *E2F3* with *MYCN* amplification or age of diagnosis in neuroblastoma. **a** Pediatric neuroblastoma patients were divided into four sub-groups based on the expression levels of *E2F3* and *MYCN* amplification. Different overall survival in each sub-group of pediatric neuroblastoma patients was determined using the Kaplan–Meier survival analysis in TARGET, GSE16476, GSE85047 and E-MTAB-1781 datasets. **b** Pediatric neuroblastoma patients were divided into four sub-groups based on the expression levels of *E2F3* and age of diagnosis. Different overall survival in each sub-group of pediatric neuroblastoma was determined

Similarly with *E2F1*, *E2F3* was also additively predicted the clinical overall survival of neuroblastoma with *MYCN* amplification or age of diagnosis. Pediatric neuroblastoma patients without *MYCN* amplification and with lower *E2F3* expression levels had significantly longer event free survival and overall survival in GSE16476, GSE85047 and E-MTAB-1781 datasets (Fig. 6a and supplementary Fig. 3a).

Furthermore, pediatric neuroblastoma patients with age at diagnosis < 18 months and with lower *E2F3* expression levels had the best clinical event free survival and overall survival than other sub-groups in TARGET, GSE16476, GSE85047 and E-MTAB-1781 datasets (Fig. 6b and supplementary Fig. 3b). On the contrary, pediatric neuroblastoma patients with age at diagnosis ≥ 18 months and with higher *E2F3* expression levels had the worst clinical event free survival and overall survival than other sub-groups (Fig. 6b and supplementary Fig. 3b).

### Construction of the risk models in pediatric neuroblastoma based on *E2F1*, *E2F3* expressions and age of diagnosis

**Fig. 7:**
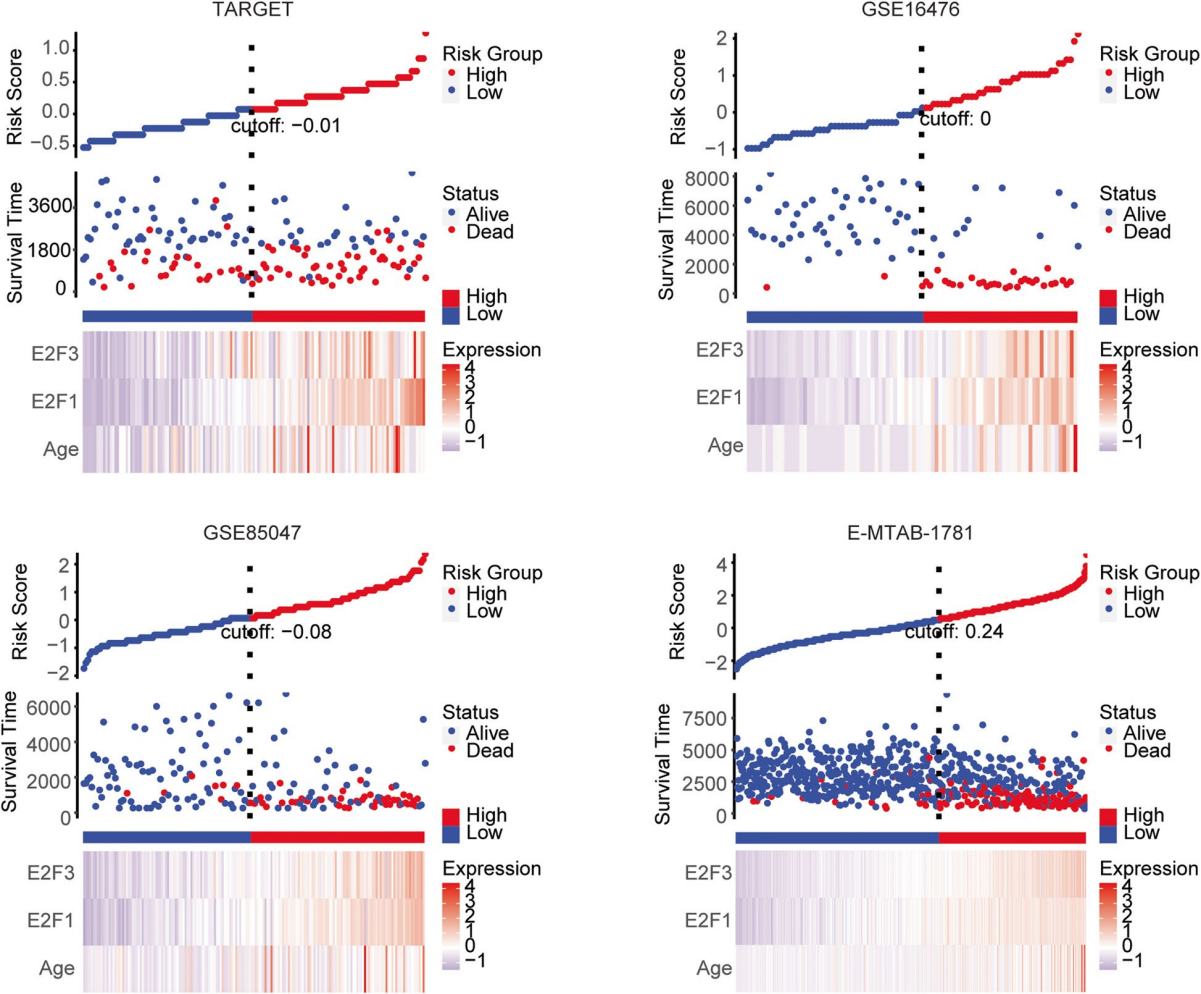
Construction of the risk models in pediatric neuroblastoma based on *E2F1*, *E2F3* expressions and age of diagnosis. The distribution of risk score, survival status and expression levels of *E2F1*, *E2F3* expressions and age of diagnosis between low-risk group and high-risk group of pediatric neuroblastoma patients in TARGET, GSE16476, GSE85047 and E-MTAB-1781datasets

We then constructed a risk model based on *E2F1*, *E2F3* expression features and age of diagnosis to predict the clinical overall survival of pediatric neuroblastoma. The risk score of each pediatric neuroblastoma patient in TARGET, GSE16476, GSE85047 and E-MTAB-1781 datasets was obtained using RiskScore formula (Fig. 7). With the increase of the risk score, the death of pediatric neuroblastoma patients was increased in TARGET, GSE16476, GSE85047 and E-MTAB-1781 datasets (Fig. 7). Moreover, the expression levels of *E2F1* and *E2F3* were positively correlated with the risk score in pediatric neuroblastoma patients (Fig. 7).

### Identification of the genes associated with *E2F1* or *E2F3*

**Fig. 8:**
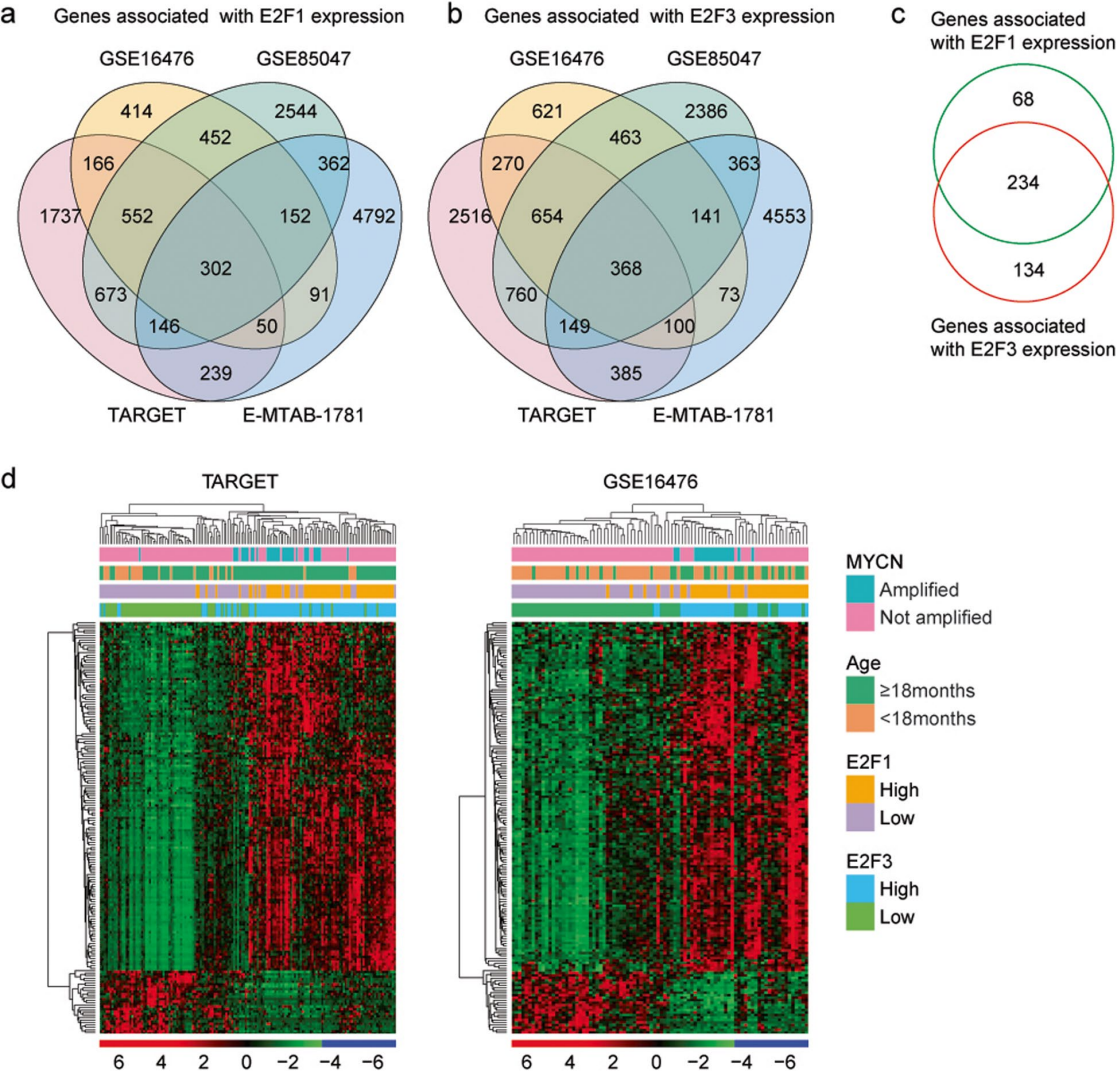
Identification of the genes associated with *E2F1* or *E2F3.* **a** Venn diagram showed the overlapped genes associated with *E2F1* expressions in TARGET, GSE16476, GSE85047 and E-MTAB-1781 datasets. **b** Venn diagram showed the overlapped genes associated with *E2F3* expressions. **c** Genes both associated with *E2F1* and *E2F3* expressions. **d** Clustering heatmaps showed the genes both associated with *E2F1* and *E2F3* expressions in TARGET and GSE16476 datasets

To further analyze the prognostic effects of *E2F1* or *E2F3* in neuroblastoma, genes differentially expressed in pediatric neuroblastoma patients with higher *E2F1* or *E2F3* expression levels were identified. Compared with neuroblastoma patients with lower *E2F1* expressions, 3865 genes were differentially expressed in pediatric neuroblastoma patients with higher *E2F1* expressions in TARGET dataset (Fig. 8a). Moreover, 2179, 5183 and 6143 genes were significantly changed in neuroblastoma patients with higher *E2F1* expressions in GSE16476, GSE85047 and E-MTAB-1781 datasets (Fig. 8a). Overlapping the differentially expressed genes showed that 302 genes were associated with the *E2F1* expressions in TARGET, GSE16476, GSE85047 and E-MTAB-1781 datasets (Fig. 8a).

Compared with neuroblastoma patients with lower *E2F3* expressions, 5202, 2690, 5284 and 6132 genes were significantly changed in neuroblastoma patients with higher *E2F3* expressions in TARGET, GSE16476, GSE85047 and E-MTAB-1781 datasets, respectively (Fig. 8b). 368 genes were associated with the *E2F3* expression in TARGET, GSE16476, GSE85047 and E-MTAB-1781 datasets (Fig. 8b). *E2F1* and *E2F3* had similar regulatory features. 234 genes were both associated with the *E2F1* and *E2F3* expressions (Fig. 8c).

The expression levels of those 234 genes associated with *E2F1* and *E2F3* expressions were further demonstrated in TARGET and GSE16476 datasets. Those genes were correlated with *MYCN* amplification and age at diagnosis of the neuroblastoma patients (Fig. 8d). Also, most of the genes were highly expressed in neuroblastoma patients with higher *E2F1* and *E2F3* expressions (Fig. 8d).

### Genes associated with the *E2F1* and *E2F3* expressions are enriched in cell cycle signaling pathway

**Fig. 9:**
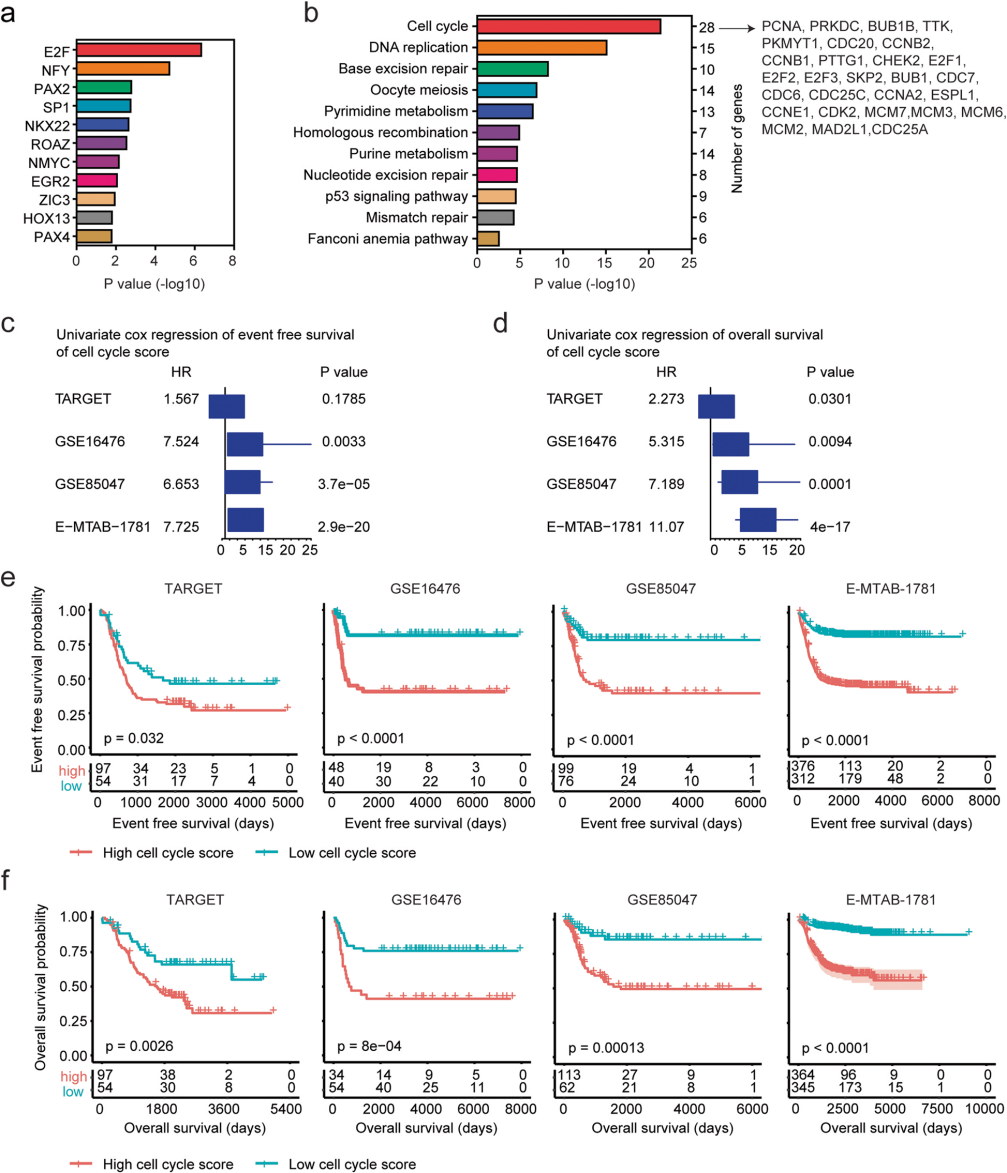
Genes associated with the *E2F1* and *E2F3* expressions are enriched in cell cycle signaling pathway. **a** Transcription factors associated with *E2F1* and *E2F3* expressions. **b** Signaling pathways associated with the *E2F1* and *E2F3* expressions. Genes enriched in cell cycle signaling pathway were showed. **c** Forest plots showed the associations of cell cycle signaling pathway with the neuroblastoma event free survival in TARGET, GSE16476, GSE85047 and E-MTAB-1781 datasets. **d** Forest plots showed the associations of cell cycle signaling pathway with the neuroblastoma overall survival. **e** The Kaplan–Meier curves showed the different event free survival of pediatric neuroblastoma patients with higher or lower cell cycle signaling pathway in TARGET, GSE16476, GSE85047 and E-MTAB-1781 datasets. **f** Different overall survival of pediatric neuroblastoma patients with higher or lower cell cycle signaling pathway in TARGET, GSE16476, GSE85047 and E-MTAB-1781 datasets

Transcription factor enrichment analysis showed that those 234 genes were associated with E2F and NFY transcription factors (Fig. 9a). Moreover, those genes were significantly correlated with cell cycle signaling pathway (Fig. 9b). 28 genes out of the 234 genes were enriched in cell cycle signaling pathway, including *E2F1*, *E2F2 E2F3* and *SKP2* genes (Fig. 9b). Previously, *SKP2* was determined as a prognostic factor of high risk neuroblastoma independent of *MYCN* status [29]. Genes associated with *E2F1* and *E2F3* expressions were also enriched in pyrimidine metabolism, purine metabolism and *TP53* signaling pathways (Fig. 9b).

The score of cell cycle signaling pathway was further calculated using ssGSEA assay. In GSE16476, GSE85047 and E-MTAB-1781 datasets, the scores of cell cycle signaling pathway were significantly correlated with the event free survival of neuroblastoma (Fig. 9c). Moreover, the scores of cell cycle signaling pathway were significantly associated with the overall survival of neuroblastoma in TARGET, GSE16476, GSE85047 and E-MTAB-1781 datasets (Fig. 9d). Patients with lower cell cycle signaling pathway had prolonged event free survival in TARGET, GSE16476, GSE85047 and E-MTAB-1781 datasets (Fig. 9e). Higher scores of cell cycle signaling pathway were also associated with the worse overall survival of neuroblastoma in TARGET, GSE16476, GSE85047 and E-MTAB-1781 datasets (Fig. 9f).

## Discussion

Neuroblastoma is a heterogeneous disease, and studies from distinct neuroblastoma cohorts may result different conclusions. Integrated analysis across different neuroblastoma cohorts may provide more robust and consistent results. In this study, using four neuroblastoma datasets, we showed that, compared with other E2F factors, *E2F1* and *E2F3* shared similar expressions and prognosis in pediatric neuroblastoma. Higher expression levels of *E2F1* or *E2F3* were associated with the worse prognosis of neuroblastoma. Also, *E2F1* and *E2F3* were associated with *MYCN* amplification and age of neuroblastoma diagnosis. Moreover, *E2F1* and *E2F3* were independent neuroblastoma prognostic factors. Combinations of *E2F1* expression, *E2F3* expression with *MYCN* amplification or age of diagnosis achieved better prognosis in neuroblastoma. On the contrary, other E2F transcription factors had no similar prognostic effect in neuroblastoma.

Furthermore, *E2F1* and *E2F3* also shared similar downstream transcriptional features in pediatric neuroblastoma. Genes associated with *E2F1* high expressions were also associated with *E2F3* high expressions. Those results suggested the functional redundancy of *E2F1* and *E2F3* in the regulation of neuroblastoma development. Similarly, in liver cancer, *E2F1* and *E2F3* were over-expressed, and synergistically induced the spontaneous development of liver cancer in mice [30, 31]. In the further development of therapeutic strategies of neuroblastoma by targeting on E2F transcription factors, *E2F1* and *E2F3* should be simultaneously inhibited to achieve better clinical outcomes.

Uncontrolled cell cycle and DNA replication are important hallmarks of cancer [32, 33]. The abnormal expressions of *E2F1* and *E2F3* may confer the uncontrolled cell cycle and DNA replication in neuroblastoma [34]. We also showed that higher scores cell cycle signaling pathway were associated with the worse overall survival of neuroblastoma. *MYCN* amplification mediated the reprogramming of metabolism is another hallmark of neuroblastoma [35–37]. Our results suggested that genes associated with *E2F1* and *E2F3* expressions were enriched in pyrimidine metabolism and purine metabolism signaling pathways. Previously, our results had shown that pyrimidine metabolism signaling pathway was associated with the progression of LUAD [38]. It was interesting to determine the prognosis of pyrimidine and purine metabolism signaling pathways in neuroblastoma. Moreover, the mechanisms of *E2F1* and *E2F3* in the regulations of metabolism signaling pathways in neuroblastoma should be further studied.

Except *MYCN* amplification and age of diagnosis, more reliable prognostic markers are required to predict the clinical outcomes of neuroblastoma [39]. In this paper, we highlighted that *E2F1* and *E2F3* were prognostic makers of neuroblastoma independent of *MYCN* amplification and age of diagnosis. The prognostic effects of *E2F1* and *E2F3* may relate to the regulations of cell cycle signaling pathway and metabolism signaling pathways. However, those conclusions were generated from published datasets and lack of validations using further experiments. Therefore, functions of *E2F1* and *E2F3* should be further studied in neuroblastoma cells by using in vivo or in vitro experiments.

## Conclusions

*E2F1* and *E2F3* were prognostic factors in neuroblastoma, independent of *MYCN* amplification and age of diagnosis. The expression levels of *E2F1* and *E2F3* were higher in neuroblastoma patients with *MYCN* amplification or age at diagnosis ≥ 18 months. Combinations of *E2F1* expression, *E2F3* expression with *MYCN* amplification or age of diagnosis achieved better prognosis of neuroblastoma. Genes associated with *E2F1* and *E2F3* expressions in neuroblastoma were significantly enriched in cell cycle signaling pathway. Higher scores of cell cycle signaling pathway were correlated with the adverse prognosis of neuroblastoma.

## Supplementary Information

**Additional file 1:** **Supplementary Figure 1.** (a) The detailed four independent datasets used in this study. (b) Different event free survival or overall survival of pediatric neuroblastoma patients in TARGET, GSE16476, GSE85047 and E-MTAB-1781 datasets. **Supplementary Figure 2.** Additively prognostic effects of E2F1 with MYCN amplification or age of diagnosis in neuroblastoma. (a) Different event free survival in each sub-group of pediatric neuroblastoma based on the expression levels of E2F1 and MYCN amplification in TARGET, GSE16476, GSE85047 and E-MTAB-1781 datasets. (b) Different event free survival in each sub-group of pediatric neuroblastoma based on the expression levels of E2F1 and age of diagnosis was determined. **Supplementary Figure 3.** Additively prognostic effects of E2F3 with MYCN amplification or age of diagnosis in neuroblastoma. (a) Different event free survival in each sub-group of pediatric neuroblastoma based on the expression levels of E2F3 and MYCN amplification in TARGET, GSE16476, GSE85047 and E-MTAB-1781 datasets. (b) Different event free survival in each sub-group of pediatric neuroblastoma based on the expression levels of E2F3 and age of diagnosis was determined.

## Data Availability

The datasets analyzed during the current study are available in the TARGET datasets, GEO datasets with accession numbers GSE16476 and GSE85047, European ArrayExpress datasets with accession numbers E-MTAB-1781 repository.

## Abbreviations

TARGET: Therapeutically Applicable Research to Generate Effective Treatments
GEO: Gene Expression Omnibus
LIHC: Liver hepatocellular carcinoma
LUAD: Lung adenocarcinoma
ssGSEA: Single sample Gene Set Enrichment Analysis
HR: Hazard ratio
KEGG: Kyoto Encyclopedia of Gens and Genome
DAVID: The Database for Annotation, Visualization and Integrated Discovery
