# Supplementary material for: Prognostic analysis of E2F transcription factors E2F1 and E2F3 in four independent pediatric neuroblastoma cohorts
Source: BMC Pediatr. 2022 Jun 29;22:376. doi: 10.1186/s12887-022-03424-w (PMC9241263; doi:10.1186/s12887-022-03424-w)
Supplement: Supplementary file 1 — Additional file 1: Supplementary Figure 1. (a) The detailed four independent datasets used in this study. (b) Different event free survival or overall survival of pediatric neuroblastoma patients in TARGET, GSE16476, GSE85047 and E-MTAB-1781 datasets. Supplementary Figure 2. Additively prognostic effects of E2F1 with MYCN amplification or age of diagnosis in neuroblastoma. (a) Different event free survival in each sub-group of pediatric neuroblastoma based on the expression levels of E2F1 and MYCN amplification in TARGET, GSE16476, GSE85047 and E-MTAB-1781 datasets. (b) Different event free survival in each sub-group of pediatric neuroblastoma based on the expression levels of E2F1 and age of diagnosis was determined. Supplementary Figure 3. Additively prognostic effects of E2F3 with MYCN amplification or age of diagnosis in neuroblastoma. (a) Different event free survival in each sub-group of pediatric neuroblastoma based on the expression levels of E2F3 and MYCN amplification in TARGET, GSE16476, GSE85047 and E-MTAB-1781 datasets. (b) Different event free survival in each sub-group of pediatric neuroblastoma based on the expression levels of E2F3 and age of diagnosis was determined. [file 12887_2022_3424_MOESM1_ESM.docx]

Supplementary data


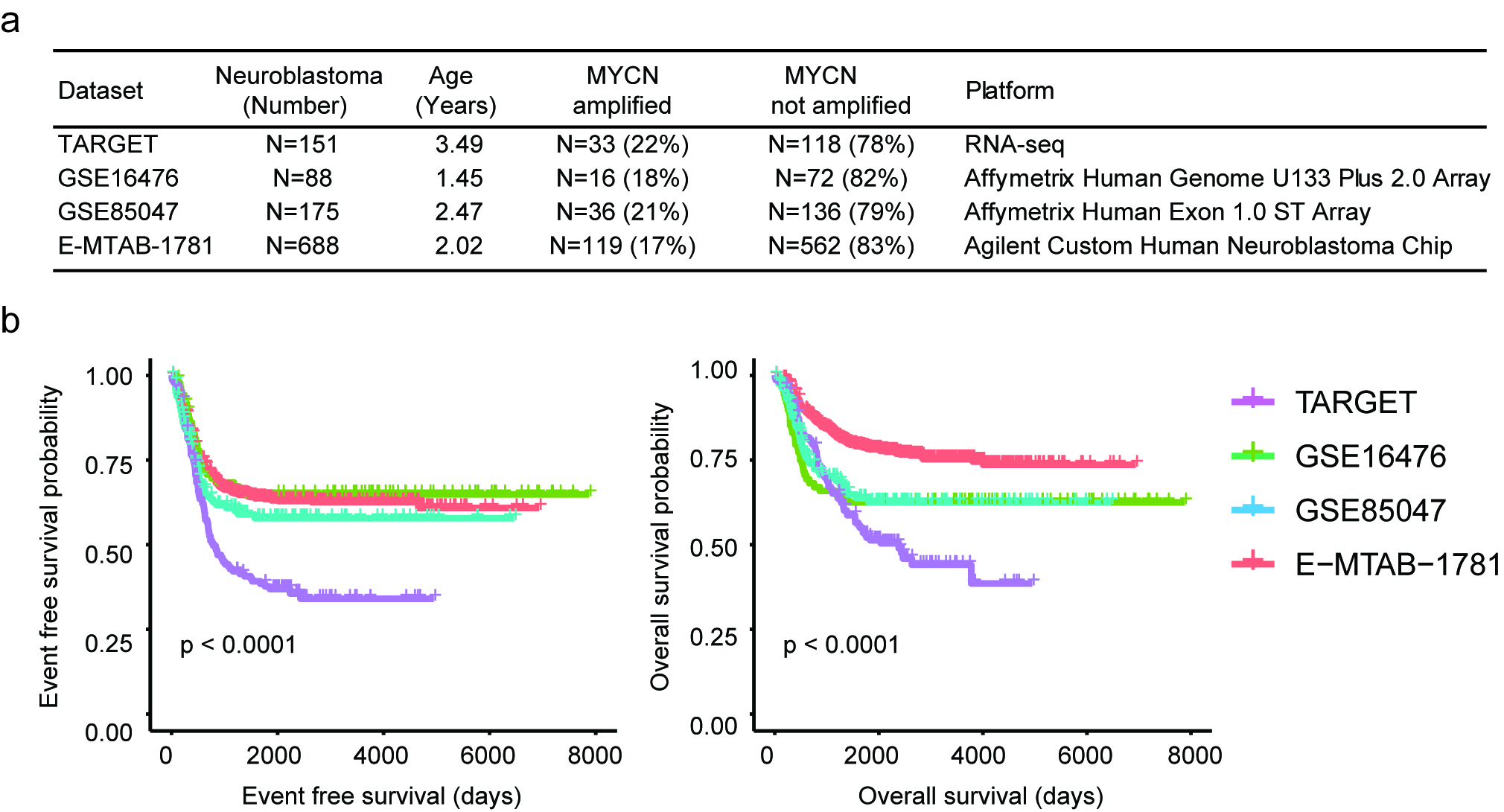


Supplementary Figure.1 (a) The detailed four independent datasets used in this study. (b) Different event free survival or overall survival of pediatric neuroblastoma patients in TARGET, GSE16476, GSE85047 and E-MTAB-1781 datasets.


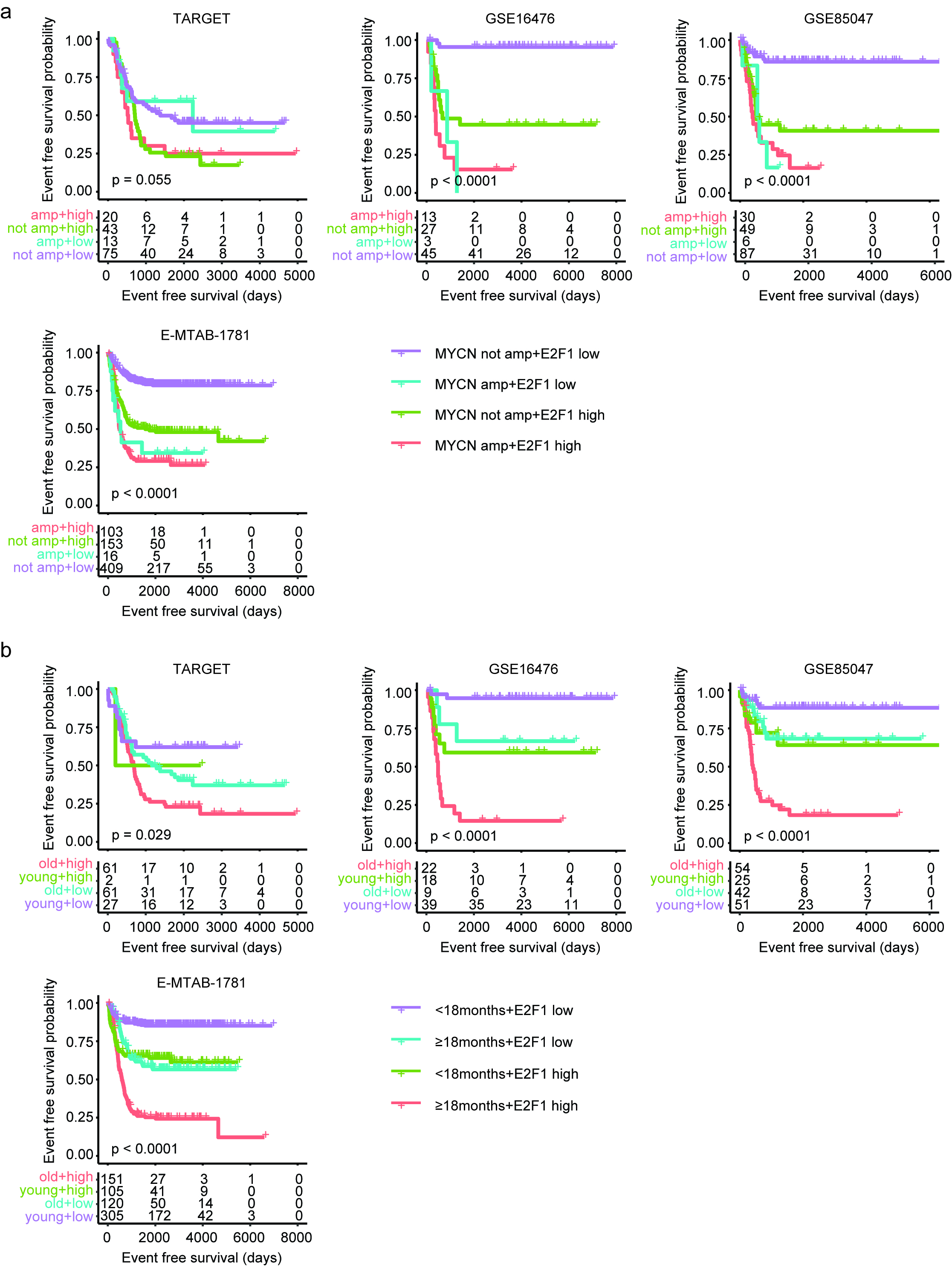


Supplementary Figure.2 Additively prognostic effects of *E2F1* with *MYCN* amplification or age of diagnosis in neuroblastoma.

(a) Different event free survival in each sub-group of pediatric neuroblastoma based on the expression levels of *E2F1* and *MYCN* amplification in TARGET, GSE16476, GSE85047 and E-MTAB-1781 datasets. (b) Different event free survival in each sub-group of pediatric neuroblastoma based on the expression levels of *E2F1* and age of diagnosis was determined.


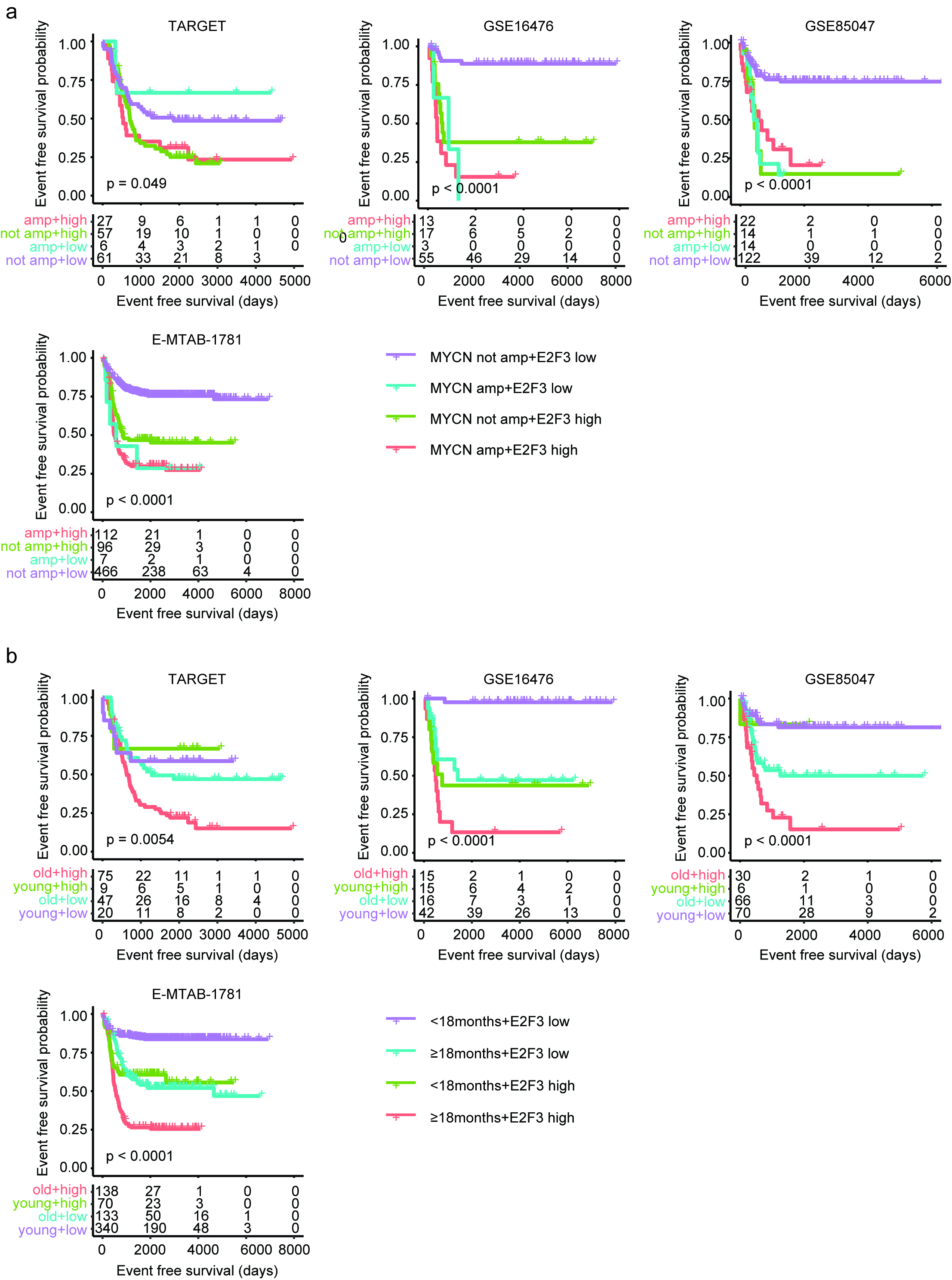


Supplementary Figure.3 Additively prognostic effects of *E2F3* with *MYCN* amplification or age of diagnosis in neuroblastoma.

(a) Different event free survival in each sub-group of pediatric neuroblastoma based on the expression levels of *E2F3* and *MYCN* amplification in TARGET, GSE16476, GSE85047 and E-MTAB-1781 datasets. (b) Different event free survival in each sub-group of pediatric neuroblastoma based on the expression levels of *E2F3* and age of diagnosis was determined.
